# Aberrant Cholesterol Metabolism in Ovarian Cancer: Identification of Novel Therapeutic Targets

**DOI:** 10.3389/fonc.2021.738177

**Published:** 2021-11-08

**Authors:** Jiangnan He, Michelle K.Y. Siu, Hextan Y. S. Ngan, Karen K. L. Chan

**Affiliations:** Departments of Obstetrics and Gynaecology, Li Ka Shing (LKS) Faculty of Medicine, The University of Hong Kong, Pok Fu Lam, Hong Kong, SAR China

**Keywords:** cholesterol metabolism, tumor microenvironment, carcinogenesis, therapeutic targets, ovarian cancer

## Abstract

Cholesterol is an essential substance in mammalian cells, and cholesterol metabolism plays crucial roles in multiple biological functions. Dysregulated cholesterol metabolism is a metabolic hallmark in several cancers, beyond the Warburg effect. Reprogrammed cholesterol metabolism has been reported to enhance tumorigenesis, metastasis and chemoresistance in multiple cancer types, including ovarian cancer. Ovarian cancer is one of the most aggressive malignancies worldwide. Alterations in metabolic pathways are characteristic features of ovarian cancer; however, the specific role of cholesterol metabolism remains to be established. In this report, we provide an overview of the key proteins involved in cholesterol metabolism in ovarian cancer, including the rate-limiting enzymes in cholesterol biosynthesis, and the proteins involved in cholesterol uptake, storage and trafficking. Also, we review the roles of cholesterol and its derivatives in ovarian cancer and the tumor microenvironment, and discuss promising related therapeutic targets for ovarian cancer.

## 1 Introduction

Ovarian cancer is one of the most aggressive malignancies worldwide (1). Due to the lack of obvious symptoms of early-stage ovarian cancer, newly diagnosed patients often present in advanced stages of disease, leading to the designation “silent killer” (2). Epithelial ovarian cancer can be classified into type I and type II ovarian tumors mainly on the basis of their cellular morphology and genetic alterations (3). Type I tumors consist of low grade serous, endometrioid, clear cell, and mucinous carcinomas, which are genetically characterized by BRAF, Kras, PTEN, or PI3KCA mutations primarily affecting PI3K/AKT/mTOR signaling (4–7). However, type II tumors mainly include high grade serous and undifferentiated carcinomas, typically with TP53 mutation and BRCA1/2 mutation (3, 8).

Metabolism in ovarian cancer shows heterogeneity, because the viability of ovarian cancer cells is maintained in a manner dependent not solely on metabolism but on the outside environment. Accumulating evidence indicates not only the active expression of aerobic glycolysis or oxidative phosphorylation (OXPHOS) in ovarian cancer but also aberrant lipid metabolism, which is strongly associated with ovarian cancer progression (9–12).

Patients with late-stage disease commonly display tumor metastases with an accumulation of ascites. The tumor microenvironment (TME) in ovarian cancer is composed of non-malignant cells, mainly including cancer-associated fibroblasts (CAF), cancer-associated adipocytes (CAA), immune-related cells, malignant cells, and secreted cytokines or other soluble molecules in ascites, which facilitate immunosuppression through crosstalk interactions among one another (13). Given that the major site of metastasis is the omentum, the TME in ovarian cancer is different from that in other cancers and is characterized as an adipocyte- and lipid-rich milieu, which has been shown to contribute to tumorigenesis, tumor immune escape, chemoresistance, and cancer recurrence (13–15). Other typical features of the tumor microenvironment include an insufficient supply of glucose and oxygen, which are non-beneficial for survival of tumor cells. To overcome this limitation, tumor cells and tumor-associated cells act in concert to develop reprogrammed adaptive metabolism (16). Ovarian tumor cells in this lipid-rich environment also tend to predominantly utilize lipid-dominant and alternative metabolic pathways (17). In addition, studies using co-culture of adipocytes and ovarian tumor cells have indicated that adipocytes promote tumor growth and metastasis of ovarian tumors, on the basis of the stimulation of adipocytes by the altered lipid metabolism in ovarian cancer, thus resulting in upregulation of lipid uptake from adipocytes and lipolysis in ovarian cancer cells (14).

Fatty acids and cholesterol are two main types of lipids. Multiple fatty acids and enzymes involved in fatty acid metabolism, such as fatty acid-binding protein 4 (FABP4), CD36 and stearoyl-CoA desaturase 1 (SCD1), significantly enhance ovarian cancer proliferation, survival, drug resistance and metastasis, and even contribute to stemness maintenance (14, 18–21). Recently, considerable evidence supporting the importance of reprogrammed cholesterol metabolism in ovarian cancer has been reported. Highly expressed proteins and enzymes involved in cholesterol metabolism promote ovarian cancer progression; cholesterol and its derivatives also contribute to proliferation and chemoresistance in ovarian cancer and have roles in the immunosuppressive tumor microenvironment (22–25). Here, we have systematically summarized the most recent findings on cholesterol and its derivatives in ovarian cancer, with the aim of comprehensively understanding their specific functions to facilitate the identification of novel markers and therapeutic targets.

## 2 Overview of Cholesterol Metabolism

**Figure 1:**
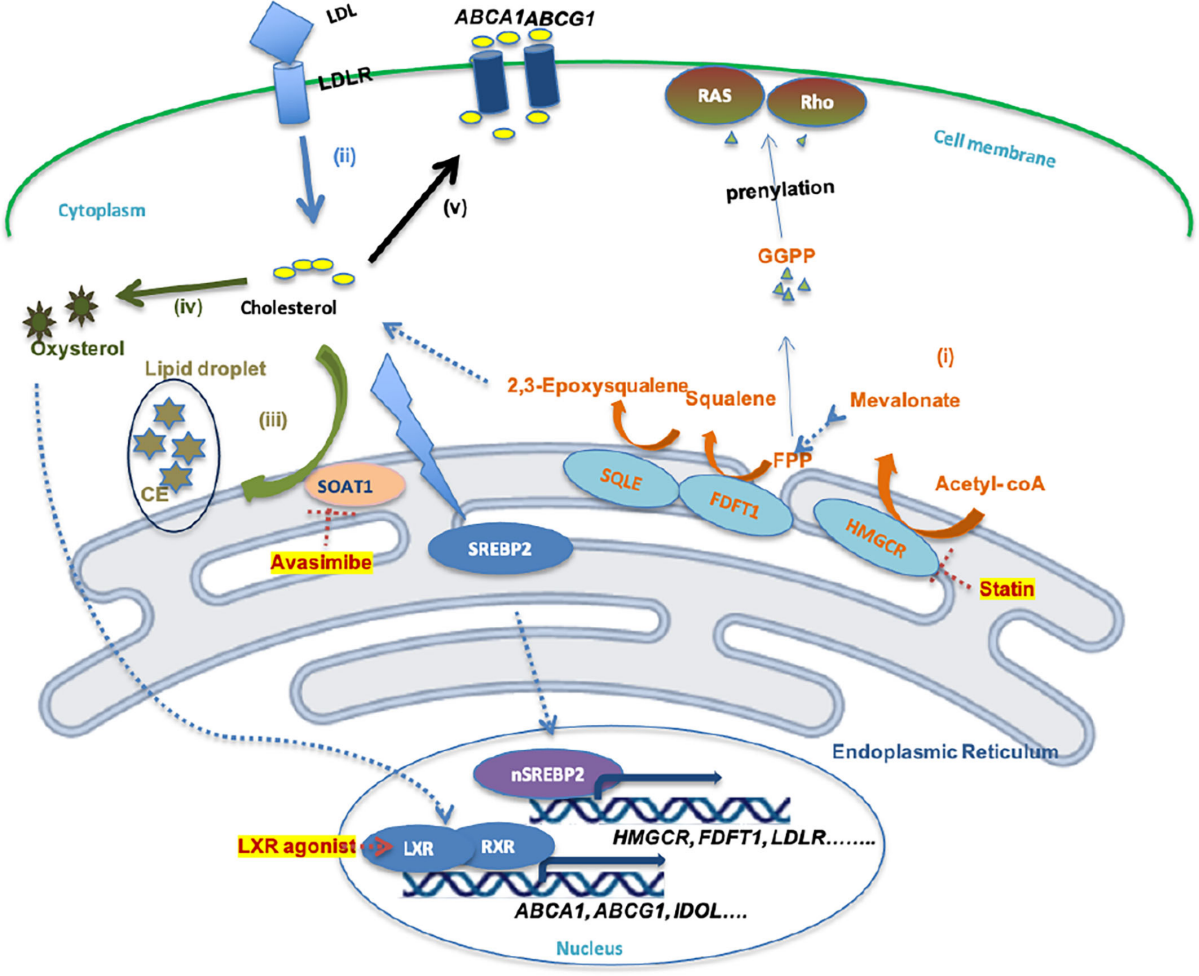
Schematic illustration of cholesterol metabolism homeostasis and potential drugs. (i)Cholesterol bio synthesis. (ii) Cholesterol uptake. (iii) Cholesterol storage. (iv) Cholesterol conversion. (v) Cholesterol efflux. (i) *De novo* cholesterol synthesis involves nearly 30 enzymatic reactions, in which HMGR and SQLE are two key rate-limiting enzymes. FPP and GGPP, intermediates in this process, contribute to the prenylation of RAS and Rho proteins, which is necessary for RAS and Rho signaling activation. (ii) Cholesterol uptake is mediated by LDL-LDLR binding, which is followed by endocytosis of LDL by cells. However, high cholesterol accumulation leads to intracellular lipo-toxicity. High intracellular cholesterol levels suppress SREBP2 transcription factor activity, thereby restricting the expression of enzymes involved in cholesterol synthesis or cholesterol uptake. (iii) Excess cholesterol is converted into cholesterol ester by SOAT1 enzyme, then stored in lipid droplets. (iv) Excess cholesterol is converted to oxysterol through multiple enzymatic or non-enzymatic process. (v) Oxysterol activates LXR-RXR signaling and results in expression of ABCA1, ABCG1, and IDOL, which promote the cholesterol efflux pathway.

Cholesterol is a fundamental metabolite of mammalian cells to maintain structural integrity and fluidity of the plasma membrane, and regulates cells or cell-to-cell interactions by mediating alterations in signaling involved in cell proliferation, immunity, and inflammation (26). Several routes of cholesterol metabolism within cells have been determined (**Figure 1**), including (i) *de novo* cholesterol synthesis, (ii) exogenous cholesterol uptake, (iii) cholesterol storage, (iv) cholesterol conversion, and (v) cholesterol trafficking (27).

(i) *De novo* cholesterol synthesis is initiated from acetyl-CoA *via* a complex enzymatic process. Within these reactions, 3-hydroxy-3-methylglutaryl-CoA (HMG-CoA) reductase (HMGCR), farnesyl-diphosphate farnesyltransferase 1 (FDFT1) and squalene epoxidase (SQLE) are key rate-limiting enzymes that convert HMG-CoA to mevalonate and squalene to 2,3-epoxysqualene (27). HMGCR, FDFT1 and SQLE are transcriptionally regulated by sterol regulatory element-binding protein 2 (SREBP2) (28). (ii) Mammalian cells take up exogenous cholesterol *via* low-density lipoprotein (LDL)-LDL receptor (LDLR) interactions, which internalizes cholesterol *via* endocytosis (12). However, free intracellular cholesterol levels require stringent control within the cytoplasm, because high levels lead to lipo-toxicity (26). An increased free cholesterol concentration >5% activates binding of SREBP cleavage-activating protein (SCAP) and Insig-1 on the endoplasmic reticulum (ER) membrane, leading to the retention of the SCAP-SREBP complex in the ER and preventing cholesterol/fatty acid synthesis and transportation, and thus lipid toxicity (29). (iii) Sterol O-acyltransferase (SOAT) is allosterically activated by elevated intracellular free cholesterol levels, promoting the conversion of cholesterols to cholesterol esters (CE), which is stored in lipid droplets (LD) (30). (iv) Oxysterol from excess cholesterol as a ligand directly activates the liver X receptor (LXR) transcription factor to regulate the (v) cholesterol efflux pathway by mediating the expression of the ATP-binding cassette (ABC) transporters, such as ABCA1 and ABCG1 (31). Excess cholesterol is exported outside the cell by ABC transporters at the cell surface, among which ABCA1 and ABCG1 are ubiquitously expressed in human cells (32). The cholesterol exported by ABCA1 is loaded onto lipid-free apolipoprotein A-I, thus producing nascent high-density lipoprotein (HDL), which in turn is converted into mature HDL by lecithin:cholesterol acyltransferase (LCAT) in the plasma (33). However, cholesterol exported by ABCG1 can directly become mature HDL (33), which can be ingested by liver cells or steroidogenic cells *via* binding to the HDL receptor, Scavenger receptor type B1 (SR-B1), thus resulting in selective CE uptake for subsequent synthesis of bile salts or steroid hormones (33, 34).

## 3 Proteins Involved in Cholesterol Metabolism in Ovarian Cancer

Several abnormally expressed proteins mediate cholesterol metabolism alterations to promote tumor cell viability, proliferation, migration, and invasion in ovarian cancer (**Table 1**). Therefore, the development of strategies targeting such proteins could lay the foundation for novel therapeutic treatment options.

**Table 1:** The roles of enzymes and proteins involved in cholesterol metabolism in ovarian cancer.

Pathway of cholesterol metabolism	Involved enzyme or protein	Expression	Role in ovarian cancer	References
Cholesterol synthesis	HMGCR	Upregulated	Enhances ovarian cancer proliferation by activating Rho/Ras signaling	(35–37)
	FDFT1	Upregulated	Contributes to chemoresistance	(23)
	SQLE	Upregulated	High expression correlates with poor-progression-free survival and overall survival rates in patients with ovarian cancer	(38)
	SREBP2	Upregulated	Contributes to chemoresistance; enhances ovarian tumor progression *via* SIK2 and MIEF2-activated PI3K/AKT/mTOR signaling	(23, 39, 40)
Cholesterol uptake	LDLR	Upregulated	Enhances chemoresistance through the LDLR/LPC/FAM83B/FGFR axis	(23, 41)
Cholesterol storage	SOAT 1	Upregulated	Promotes ovarian tumor progression, SOAT1 inhibition impaired tumor cell proliferation, migration and increased chemosensitivity	(42)
Cholesterol trafficking	ABCA1	Upregulated	Promotes tumor cell proliferation, migration and invasion, chemoresistance and stemness maintenance	(43, 44)
	ABCG1	Upregulated	Upregulated in only high grade serous ovarian cancer; further research needed	(45)
	LXR	————	Inhibition of ovarian tumor proliferation after LXR agonist treatment	(46)
	SR-B1	Upregulated	High expression positively associated with patient survival rates	(38)

HMGCR, 3-hydroxy-3-methylglutaryl-CoA (HMG-CoA) reductase; FDFT1, Farnesyl-Diphosphate Farnesyltransferase 1; SQLE, Squalene epoxidase; SREBP2, Sterol regulatory element-binding protein 2; LDLR, Low-density lipoprotein receptor; SOAT 1, Sterol O-acyltransferase; LXR, Liver X receptor (LXR); ABCA1, ABC subfamily A member 1; ABCG1, ABC subfamily G member 1; SR-B1, Scavenger receptor type B1.

### 3.1 Cholesterol Biosynthesis

#### 3.1.1 HMG-CoA Reductase (HMGCR)

HMGCR, a glycoprotein located in the ER, is one of rate-limiting enzymes in the mevalonate pathway that catalyzes the generation of mevalonate from HMG-CoA with the consumption of two NAPDH molecules (26, 47). In addition to cholesterol generation to meet nutritional and membrane structure demands, intermediates of the mevalonate pathway are essential for the regulation of well-characterized oncogene-mediated signaling molecules, such as farnesyl pyrophosphate (FPP) and geranylgeranyl pyrophosphate (GGPP), which are essential for prenylation of the small GTPase proteins, Ras and Rho (48). Prenylation of Ras or Rho is critical for their membrane localization and activity (49). Oncogenic roles of HMGCR have been reported in various tumor types, including gastric, liver, and breast cancers (50–53).

The Keto and Wolf groups have reported higher HMGCR expression in cell lines and primary cultures from ovarian cancer than in normal ovarian epithelial cell lines and ovarian tissues (35, 54). Immunohistochemical expression was observed in the majority of ovarian cancer tissues (55). Mechanistically, gain-of-function TP53 variants displayed ectopic expression in SKOV-3, while the lack of endogenous p53 expression or native TP53 mutations in OVCAR-3 resulted in elevated HMGCR mRNA levels and protein expression (36, 54). HMGCR inhibition with specific statin-like drugs has been found to inhibit monolayer and ovarian tumor spheroid cellular proliferation and tumor growth in xenograft mouse models, enhance autophagy, induce cellular arrest in G0/G1, promote extrinsic and mitochondrial (intrinsic) apoptosis with increased activity of caspase-3, 8, 9 and elevated Poly (ADP-ribose) polymerase (PARP) cleavage, and increase the sensitivity of ovarian cancer cells to carboplatin (35, 37, 56, 57). However, the addition of GGPP or mevalonate instead of cholesterol rescued the anti-proliferative effect mediated by statin and activated Ras/Rho signaling (35, 37).

#### 3.1.2 Farnesyl-Diphosphate Farnesyltransferase 1 (FDFT1)

FDFT1, also known as squalene synthase, is located in the ER and acts downstream of HMGCR to synthesize squalene from FPP (58). The role of FDFT1 in cancer development is ambiguous at present. Reports to date suggest oncogenic effects of FDFT1, such as promoting proliferation, increasing anti-apoptotic protein levels, and preventing ferroptosis by increasing squalene levels in some cancer types, and conversely, plays an anti-oncogenic role in other cancers. For instance, overexpression of FDFT1 has been shown to induce the suppression of glycolysis through the blockage of AKT/mTOR/HIF-1α signaling in colorectal cancer (59).

FDFT1 is highly expressed in ovarian cancer. Zheng et al. showed a nearly 7-fold upregulation of FDFT1 in A2780 cisplatin-resistant ovarian cancer cells relative to sensitive cells (23). Interestingly, siRNA mediated FDFT1 inhibition in A2780 cells slightly augmented tumor cell proliferation, while its overexpression impaired migration and invasion of SKOV3 and 3AO cells (60). These findings may be attributed to squalene accumulation inducing cytotoxicity within cells (61).

#### 3.1.3 Squalene Epoxidase (SQLE)

SQLE, also known as squalene monooxygenase, is located in the ER and is a rate-limiting enzyme in the mevalonate pathway that mediates the conversion of squalene into 2,3-epoxysqualene *via* usage of NADPH and a molecular oxygen (62). SQLE overexpression has been observed in multiple cancers, including breast cancer, liver and lung cancer, and is correlated with their aggressive behaviors and poorer prognosis (63–67). High expression and gain of the SQLE locus have been reported in ovarian cancer (63). Furthermore, the Kaplan-Meier analysis of ovarian cancer has shown that high SQLE expression is correlated with relatively poor progression-free survival and overall survival rates in patients with ovarian cancer (38). Therefore, further research on the potential involvement of SQLE in the pathogenesis of ovarian cancer should be considered.

#### 3.1.4 Sterol Regulatory Element-Binding Protein 2 (SREBP2)

SREBP2 is a key transcription factor of enzymes involved in cholesterol synthesis and transport, including HMGCR, FDFT1, SQLE and LDLR (28). Under conditions of cholesterol sufficiency, SREBP2 is located in the ER in an inactive state (28). Upon depletion of cholesterol, SREBP2 translocates to the Golgi apparatus and is cleaved by site 1 protease (S1P) and S2P to an active state. The active protein subsequently enters the nucleus to bind other regulatory factors at the promotor regions of target genes (68).

SREBP2 can enhance chemotherapeutic drug resistance in ovarian cancer cells *via* the upregulation of cholesterol synthesis (23). Zheng et al. (23) reported that the levels of SREBP2 and SREBP-targeted genes, such as HMGCR, FDFT1 and LDLR, in A2780 cells were proportionally correlated with cisplatin doses. Karashcuk and co-workers additionally reported that SREBP2 mediates ovarian cancer recurrence and escape from cell cycle arrest after paclitaxel treatment (69). The inhibition of SREBP2 with CRISPR technology in OVCAR-8 cell lines led to slower recovery rates of cell growth following paclitaxel treatment, compared to control cells (70). Thus, targeting of SREBP2 may improve drug sensitivity and lower the recurrence of ovarian cancer.

In addition to being regulated by the free intracellular cholesterol level, SREBP2 expression is also regulated by the PI3K/AKT/mTOR signaling pathway (71). In the cholesterol synthesis pathway, SREBP2 is upregulated by salt-inducible kinase 2 (SIK2), an AMPK-related kinase, and mitochondrial elongation factor 2 (MIEF2)-activated PI3K/AKT or ROS/AKT/mTOR signaling, thus, leading to the promotion of ovarian tumor growth (39, 40).

### 3.2 Cholesterol Uptake

#### 3.2.1 Low-Density Lipoprotein Receptor (LDLR)

Binding of LDLR, a transmembrane glycoprotein located on the cell plasma membrane, to LDL facilitates cholesterol uptake *via* endocytosis (72). Based on TCGA data, high LDLR expression is significantly associated with poor overall survival rates of ovarian tumor patients (22). IHC findings have revealed a strong intensity of LDLR in endometrioid and clear cell types of ovarian cancer (41). Zheng et al. showed that LDLR was upregulated in an ovarian cancer cell line resistant to cisplatin (23). Silencing of LDLR improved the sensitivity of ovarian tumor cells to cisplatin treatment by mediating the LPC (lysophosophatidylcholine)/FAM83B (family with sequence similarity 83 member B)/FGFR (fibroblast growth factor receptor) axis (41). Therefore, LDLR may be recognized as a marker of cisplatin treatment response to ovarian tumors, and in particular, the endometrioid and clear-cell types.

### 3.3 Cholesterol Storage

#### 3.3.1 Sterol O-Acyltransferase (SOAT)

Sterol O-acyltransferase (SOAT), also designated as acyl-coenzyme A cholesterol acyltransferase (ACAT), converts cholesterol and acyl-CoA to cholesterol esters (CE) in the ER, which are then stored in lipid droplets (33). SOAT exists as two isoforms, including SOAT1 and SOAT2. SOAT1 is generally detectable in all tissues, while SOAT2 is limited to the liver or intestinal tissue (73). SOAT1, but not SOAT2, is expressed highly in liver cancer, brain cancer, prostate cancer, and pancreatic cancer tissues and associated with their low overall survival rates (74–77). These findings suggest an oncogenic role for SOAT1 and support its utility as a potential therapeutic target.

Ayyagari et al. (42) reported higher SOAT1 expression levels than SOAT2 expression levels, and elevated CE levels in ovarian cancer cell lines compared to normal cell lines. Mechanistically, SOAT1 inhibition by shRNA or Avasimibe suppressed proliferation, migration and invasion of SKOV3, OC-314, and IGROV-1 cell lines by promoting mitochondrial apoptosis; the cells showed decreased mitochondrial potential, high activity of caspase 3/7, and increased ROS and p53 expression, regardless of mutation status. Furthermore, knockdown of SOAT1 improved the cisplatin sensitivity of ovarian cancer cells (42).

Of note, SOAT1 deficiency in CD8+ T cells augments their tumor-killing ability *via* increasing the cholesterol content on the plasma membrane and subsequently promoting T-cell receptor (TCR) clustering and immunological synapse formation in CD8+ T cells (78). SOAT1 depletion in mesothelin-directed chimeric antigen receptor T cells (CART) can strengthen their anti-tumor response against pancreatic carcinoma *in vitro* or *in vivo* (79). Therefore, SOAT1 inhibition may mediate dual anti-tumor effects in cancer treatment in terms of tumor inhibition and immunity enhancement, and is likely to have value in combination with immunotherapy.

### 3.4 Cholesterol Trafficking

#### 3.4.1 ABC Subfamily A Member 1 (ABCA1)

ATP-binding cassette (ABC) transporters in the cell membrane mainly consisting of ABC subfamily A member 1 (ABCA1) and ABC subfamily G members 1, 5 and 8 (ABCG1, ABCG5, ABCG8) contribute to cholesterol efflux (80). Unlike ABCG5 and ABCG8 that are restricted to hepatocytes and enterocytes, ABCA1 and ABCG1 are ubiquitously expressed throughout the body (81). Hedditch et al. reported that high ABCA1 expression in ovarian cancer tissue was significantly correlated with poor survival outcomes of patients. In terms of functional analyses, depletion of ABCA in A2780, 27/87 and SKOV3 ovarian cancer cell lines *via* siRNA attenuated colony formation, migration, and invasion (43). Moreover, ABCA1 promoted ovarian cancer drug resistance and tumorigenesis. Silencing of ABCA1 in MCP2 platinum-resistant cells led to improved cisplatin sensitivity (82). In addition, ABCA1 was upregulated in EPCAM+CD45+ tumor cells derived from ascites of patients with ovarian cancer with aggressive features (44). Chou et al. showed that hypermethylation of ABCA1 was correlated with a poorer prognosis of ovarian cancer patients (83). Specifically, *in vitro* treatment of MCP3 and HeyC2 cell lines with shABCA1, or an *in vivo* HeyC2 cell-based xenograft mouse model mimicking hypermethylation enhanced tumor cell growth (83). Thus, the roles of ABCA1 in ovarian cancer require further investigation.

#### 3.4.2 ATP Binding Cassette Subfamily G Member 1 (ABCG1)

ABCG1, located at the cell membrane surface, mediates cholesterol export from cells by mature HDL. High expression of ABCG1 has been observed in pancreatic cancer, breast cancer, lung cancer, and colon cancer (84–87). ABCG1 promotes cell proliferation, migration, and invasion in lung cancer cells, and is associated with expression of anti-apoptotic proteins (B-cell lymphoma 2 (BCL2) or Myeloid-cell leukemia 1 (MCL1), stemness markers (CD133 and ALDH), and proliferative markers (such as c-Myc) (87). ABCG1 inhibition by knockdown suppresses tumor growth in a colon tumor mouse model by blocking extracellular vesicle (EV) lipid efflux, thereby leading to the accumulation of EVs, which mediate cellular toxicity (86). In addition, ABCG1 is associated with tumor immunity. ABCG1 contributes to the macrophage phenotype shift from M1 to M2 (88). Macrophages with ABCG1 deficiency have higher cytotoxicity with NF-κB activation (88). In addition, depletion of ABCG1 causes hyperproliferation of CD4+ T cells in the peripheral blood in mice (89). These findings illustrate that ABCG1 may be a promising anti-tumor target. However, the high expression of ABCG1 has been observed in only high grade serous ovarian carcinoma (HGSC) (45). Its detailed mechanisms in ovarian cancer should be further explored.

#### 3.4.3 Liver X Receptor (LXR)

LXR, which belongs to the nuclear receptor family, plays an important role in maintaining intracellular cholesterol homeostasis (31). LXR is activated by LXR agonists and subsequently forms a heterodimer with retinoid X receptor (RXR). This LXR-RER heterodimer combined with co-activator binds LXR-responsive-elements (LXREs) in the nucleus and mediates the expression of cholesterol metabolism-related genes, such as ABCA1, ABCG1, and inducible degrader of LDLR (IDOL) (90). LXR activation mediates anti-tumor effects in multiple cancers (91). LXR activation induces expression of inducible degrader of LDLR (IDOL), which decreases the LDLR expression induced by EGFR/SREBP-1 signaling in glioblastoma tumor cells (92). Likewise, LXR activation by its agonists significantly suppresses ovarian tumor cell proliferation (46).

#### 3.4.4 Scavenger Receptor Type B1 (SR-B1)

SR-B1 recognizes HDL and then selectively takes up CEs into cells without the apolipoprotein moiety. SR-BI is commonly expressed in the liver cells and steroidogenic cells. SR-BI is highly expressed in multiple cancer cell lines including ovarian cancer cells lines (93). High SR-BI expression has been observed in lung cancer and breast cancer, and it is associated with malignancy and poor prognosis (94, 95). SR-BI is recognized as a biomarker of melanoma progression in patients and has been associated with STAT5 expression in clinical samples (96). However, SR-B1 expression in patients with ovarian cancer patients is positively correlated with survival rate (38). To provide further clarification, its detailed mechanisms require further exploration.

## 4 Roles of Cholesterol and Cholesterol Derivatives in Ovarian Cancer and Tumor Microenvironment

### 4.1 Cholesterol

Previous studies have shown high cholesterol levels in ascites fluid in ovarian tumors (97). Helzlsouer et al. initially reported that the cholesterol concentration in blood was proportionally correlated with the risk of ovarian cancer (98). In addition, LDL, a main blood carrier of cholesterol, and large amounts of cholesterol are associated with aggressiveness and poor survival outcomes of ovarian cancer (99). In the murine ID8 model of ovarian cancer, mice subjected to a high cholesterol diet exhibited increased tumor growth compared to that observed in the control groups (24). Dysregulated cholesterol homeostasis has been reported to enhance platinum resistance in ovarian cancer (22). Besides, high cholesterol levels in aggressive ascites were shown to contribute to cisplatin resistance in ovarian tumor cells by activating an LXR α/β nuclear receptor, with sequential upregulation of multidrug resistance protein 1 (MDR1) (100). High cholesterol loading in mitochondria perturbs mitochondrial function, inhibiting mitochondrial membrane permeabilization and the release of cytochrome c, a pro-apoptotic signal, thus contributing to chemotherapy resistance in liver cancer cells (101). The effects of dysregulated cholesterol homeostasis in mitochondria on drug resistance in ovarian cancer requires further investigation.

Cholesterol also influences energetic metabolism, thus contributing to tumor progression. In breast cancer cells, exogenous cholesterol alters metabolic pathways and consequently enhances cell proliferation in an estrogen-related receptor alpha-dependent manner, thus increasing oxidative phosphorylation and the tricarboxylic acid cycle (TCA) cycle (102). Aerobic glycolysis has been found to be augmented by exogenous cholesterol in only triple-negative breast cancer cell lines (102). Furthermore, high mitochondrial cholesterol loading increases hexokinase translocation to the mitochondria and may contribute to aerobic glycolysis in cancer cells (103). However, the relationship between cholesterol and energetic metabolism is less clear in ovarian cancer and requires further study.

Other than its effects on tumor cells, cholesterol may contribute to the immunosuppressive TME. Evidence has shown that cholesterol influences tumor-associated macrophages (TAM) in the microenvironment. Peritoneal TAMs in the ovarian cancer mouse model were reported to show increased cholesterol efflux activated by high molecular weight hyaluronic acid secretion from ID8 ovarian tumor cells, in turn, augmenting IL-4/PI3K/Akt/STAT6 signaling. However, attenuation of the IFN-γ-induced gene signature in TAM contributes to immunosuppression and the energetic needs of tumors (25). Specific knockout of ABCA1 or ABCG1 with the inhibition of cholesterol efflux in TAM effectively reversed the pro-tumorigenic effect of TAM in ovarian cancer, which could be applied to develop a novel therapeutic strategy. In addition, a large amount of cholesterol secreted from tumor cells impairs the cytotoxicity of CD8+ effector T cells and induces exhausted CD8+ T cells. HMGCR knockdown or statin treatment in B16 melanoma cells significantly decreases the frequency of exhausted CD8+ T cells at tumor sites (104). Mechanistically, high cholesterol augments endoplasmic reticulum (ER) stress in CD8+ T cells and consequently results in XBP-1 activation, which elevates the expression of immune checkpoint proteins, such as T cell immunoglobulin and mucin domain-containing protein 3 (TIM-3), Programmed cell death protein 1 (PD-1), Lymphocyte activation gene 3 protein (LAG-3), and 2B4 (CD244), in CD8+ T cells (104).

### 4.2 Oxysterol

Oxysterol, the hydroxylation product of cholesterol, participates in numerous cellular processes, such as cell signaling, membrane fluidity, and the activation of membrane proteins, similar to cholesterol (105). The 27-Hydroxycholesterol (27HC), a type of oxysterol, is catalyzed from cholesterol by Cytochrome P450 Family 27 Subfamily A Member 1 (CYP27A1) (106). High CYP27A1 expression is associated with poor prognosis at the early stages of disease and poorer progression-free survival but serves as a positive predictor in late-stage ovarian cancer (24). Functionally, exogenous 27HC treatment could abolish the proliferative capacity of ovarian cancer cell lines *via* LXR activation-induced cholesterol efflux in tumor cells. Intriguingly, however, CYP27A1 or exogenous 27HC treatment in the ovarian cancer mouse model has also been shown to augment peritoneal tumor spread and carboplatin resistance, consistent with Kaplan–Meier analyses of CYP27A1 in ovarian cancer patients (24). These data suggest that CYP27A1 and its product 27HC promote ovarian cancer progression by influencing the tumor microenvironment, rather than intrinsic effects on the tumor itself.

Reprogrammed macrophage patterns have been observed in ovarian tumors in the presence of exogenous 27HC, including increased concentrations of monocytic myeloid-derived suppressor cells (MDSC) and decreases in antigen-presenting macrophages (24). Genetic depletion of CYP27A1 could reverse the immunosuppressive effect of 27HC. In addition, a combination of the CYP27A1 inhibitor, GW273297X, and anti-PDL1 antibodies induced a significant decrease in ovarian tumor growth in mouse models compared to either treatment alone (24). In addition, the oxysterol secreted by tumor cells has been shown to impair the antigen presentation of dendritic cells (DC) by LXR-α signaling activation, thus mediating downregulation of the expression of C-C chemokine receptor type 7 (CCR7), a lymphoid homing marker of DC, on the DC cell surface (107). LXR-α activation in DCs compromises DC migration to lymph nodes, thus decreasing T cell priming (107). Treatment with Sulfotransferase 2B1b (SULT2B1b), an LXR ligand-inactive enzyme, relieves the CCR7 inhibition in DCs, and restores DC function and the anti-tumor response (107). LXR signaling activation suppresses the proliferation and expansion of T cells (108). However, Tavazoie et al. have shown that LXR activation suppressed the immunosuppressive effect of myeloid-derived suppressor cells (MDSC) by inducing Apolipoprotein E (ApoE) expression and consequently augmenting the T cell killing ability (109).

Another type of oxysterol, 25-hydroxycholesterol (25HC), is synthesized by cholesterol 25-hydroxylase (CH25H) (110). The specific role of 25-hydroxycholesterol (25HC) in ovarian cancer remains to be established. The 25HC is reported to stimulate the proliferation of BG-1 ovarian cancer cells in an estrogen receptor-α-activation-dependent manner (110). However, 25HC combined with statin reduced the viability of OVCAR-8 and SKOV3 cell lines *via* the suppression of SREBP2. SREBP2 suppression was greater following the combined treatment compared to that observed with statin treatment alone (111). Therefore, future studies should focus on the precise mechanisms of action of 25HC in ovarian cancer.

## 5 Potential Therapeutic Drugs in Ovarian Cancer

### 5.1 Statins

Statins are specific inhibitors of HMGCR that block the mevalonate pathway (112). Statins were originally used to lower the cholesterol level in blood and were found to be well-tolerated. Freed-Pastor et al. demonstrated that upregulation of the mevalonate pathway was mostly mediated by TP53 mutations, which is a dominant genetic mutation profile in ovarian cancer. Several reports have confirmed that lipophilic statins (**Table 2**), such as simvastatin and lovastatin, but not hydrophilic statins, significantly suppressed cell viability and proliferation, stemness, invasion and migration, and enhance mitochondrial apoptosis and chemotherapeutic sensitivity of ovarian cancer cell lines and primary ovarian cancer samples derived from patients or mouse models, without causing damage to normal cells (35, 37, 54, 56, 57). Statins exert pluripotent effects against cancer. Göbel and co-workers showed that lipophilic statins attenuated the expression of IL-6, IL-8, Vascular endothelial growth factor (VEGF), and Transforming growth factor beta (TGF-β), which contributed to ovarian tumor progression (115). Meanwhile, statin treatment of ovarian cancer cell lines activated c-Jun N-terminal kinase (JNK) signaling and induced the pro-apoptotic protein, Bim, reduced c-Myc phosphorylation, and blocked Ras/Rho signaling (37, 57, 113).

**Table 2:** Potential drugs for targeting cholesterol metabolism in ovarian cancer.

Involved proteins	Drug	Function	Mechanism	Cell source	Clinical trial	References
HMGCR	Atorvastatin	Inhibit proliferation, stemness, migration, invasion; enhance apoptosis, cell cycle arrest and chemotherapy sensitivity	Decrease p-S6 and p-c-Myc	Hey, SKOV3.	NCT02201381, Phase 3 (Recruiting)	(57)
	Simvastatin		Block Ras/Rho signaling *via* reduction of FPP and GGPP, attenuate stemness by inactivating Hippo/YAP/TAZ/signaling	A2780, Hey8, primary ovarian cancer cells	NCT04457089, Early Phase 1 (Recruiting)	(35, 56)
	Lovastatin		Upregulate Bim (pro-apoptotic protein) expression, induce autophagy, anti- Ras/Rho signaling	Hey1B, SKOV3, OVCAR5, mouse model	NCT00585052, Phase 2 (Terminated)	(37, 113)
SOAT1	Avasimibe	Anti-proliferative effect	———————	OC314, SKOV3, IGROV-1	—————	(42)
Liver X receptor	GW3965	Induce apoptosis	Decrease LD accumulation induced by hypoxia	IGOVR-1, SKOV3	—————	(114)
	T0901317	Anti-tumor growth, induce cell cycle arrest	Upregulate p21, p27. expression in an FXR activation dependent manner	CaOV3, A2780, SKOV3	—————	(46)

In addition to promoting resistance against tumor growth, statins are reported to enhance antigen presentation in dendritic cells (DC) and T cell cytotoxic functions in a B16 melanoma mouse model by attenuating Rab5 protein prenylation by GGPP or FPP, which are involved in the endosomal trafficking process; thus, reducing antigen internalization and degradation at the cell surface (116). Notably, the combination of statin with anti-PD1 antibodies exerted a stronger synergistic anti-tumor effect comparison with the alone treatment (116). Therefore, statin-like drugs present potent therapeutic options for ovarian cancer.

Several clinical trials (NCT04457089 and NCT00585052) on ovarian cancer have been conducted. Statins, and in particular, lipophilic statins are clearly associated with a lower risk of ovarian cancer occurrence (117).

### 5.2 Avasimibe

Avasimibe, an inhibitor of SOAT and a cholesterol-lowering drug, can suppress CE generation. Accumulating preclinical studies have revealed the inhibitory effects of Avasimibe on tumor growth in various cancer types, including hepatocellular carcinoma, glioblastoma, pancreatic adenocarcinoma, and prostate cancer through the regulation of intracellular cholesterol metabolism (74–77). Ayyagari et al. (42) demonstrated the anti-tumor growth effect of Avasimibe in ovarian cancer cell lines.

In addition to its tumor suppression activity, Avasimibe is also an immunomodulatory agent. Avasimibe can augment the tumor-killing function of CD8+ T cells *via* the enhancement of T cell receptor (TCR) signaling and immunological synapse formation of CD8+ T cells by enhancing the cholesterol level of the plasma membrane (78). Consequently, many combined therapeutic strategies have been developed for Avasimibe and other immunotherapies, such as anti-PD1 antibodies, cancer stem cell-dendritic cell (CSC-DC) vaccines, and Kras peptide vaccines (78, 118, 119).

In earlier atherosclerosis clinical trials, Avasimibe exhibited a well-tolerated safety profile (120), and thus, should be strongly considered for ovarian cancer clinical trials.

### 5.3 LXR Agonist

Liver X receptor, a nuclear receptor, senses alterations in cholesterol metabolism (33). Natural ligands of this receptor include different types of oxysterol, such as 22-hydroxycholesterol (22HC), 20-hydroxycholesterol (20HC), and 27HC (121). The 27HC is known to exert anti-proliferative effects in ovarian tumors. GW3965 and T0901317 are synthetic LRX ligands (121) and Curtarello et al. showed that GW3965 could significantly promote apoptosis of SKOV3 and IGROV1 cell lines by reducing LD accumulation under hypoxic conditions to a similar degree as the anti-VEGF antibody, bevacizumab (114). Combination treatment with GW3965 and bevacizumab further promoted treatment efficiency. Additionally, T0901317 exerted a significant inhibitory effect on ovarian cancer cells in a dose- and time-dependent manner *via* interactions with farnesoid-X receptor (FXR), rather than *via* LXR activation (46). However, limited research has focused on the involvement of LXR, and further experiments are required to determine the role of LXR agonists in ovarian cancer.

## 6 Conclusions

Numerous studies have implicated essential enzymes or proteins involved in cholesterol metabolism in ovarian cancer, thus, supporting the theory that aberrant cholesterol metabolism contributes to disease progression. Cholesterol and oxysterol, its derivative, are not only intrinsic tumor-promoting factors but also extrinsic tumor-promoting factors *via* reprogramming the tumor microenvironment. Elevated levels of cholesterol and oxysterol also contribute to the immunosuppressive environment. High cholesterol level in TME contributes to the generation of exhausted CD8+T cells. Oxysterol can reprogramme the TAM pattern and influence the antigen presentation ability of DC in the tumor site. Thus, drugs targeting cholesterol metabolism may present potential treatments and even overcome the immunotherapy resistance, as a combined therapy with immune checkpoint blockades. Further studies are needed to clarify the specific roles and associated mechanisms of action of these proteins in the pathogenesis of ovarian cancer to facilitate the development of therapeutic clinical agents.

## Author Contributions

Writing the original draft preparation, JH. Writing—review and editing, MS and KC. Supervision, MS, HN, and KC. All authors contributed to the article and approved the submitted version.

## Funding

The work was funded by the University of Hong Kong(202011159187).

## Conflict of Interest

The authors declare that the research was conducted in the absence of any commercial or financial relationships that could be construed as a potential conflict of interest.

## Publisher’s Note

All claims expressed in this article are solely those of the authors and do not necessarily represent those of their affiliated organizations, or those of the publisher, the editors and the reviewers. Any product that may be evaluated in this article, or claim that may be made by its manufacturer, is not guaranteed or endorsed by the publisher.
